# Research on Classification of Fine-Grained Rock Images Based on Deep Learning

**DOI:** 10.1155/2021/5779740

**Published:** 2021-09-20

**Authors:** Yong Liang, Qi Cui, Xing Luo, Zhisong Xie

**Affiliations:** College of Mechanical and Control Engineering, Guilin University of Technology, Guilin, Guangxi 541004, China

## Abstract

Rock classification is a significant branch of geology which can help understand the formation and evolution of the planet, search for mineral resources, and so on. In traditional methods, rock classification is usually done based on the experience of a professional. However, this method has problems such as low efficiency and susceptibility to subjective factors. Therefore, it is of great significance to establish a simple, fast, and accurate rock classification model. This paper proposes a fine-grained image classification network combining image cutting method and SBV algorithm to improve the classification performance of a small number of fine-grained rock samples. The method uses image cutting to achieve data augmentation without adding additional datasets and uses image block voting scoring to obtain richer complementary information, thereby improving the accuracy of image classification. The classification accuracy of 32 images is 75%, 68.75%, and 75%. The results show that the method proposed in this paper has a significant improvement in the accuracy of image classification, which is 34.375%, 18.75%, and 43.75% higher than that of the original algorithm. It verifies the effectiveness of the algorithm in this paper and at the same time proves that deep learning has great application value in the field of geology.

## 1. Introduction

Rocks are naturally occurring minerals or solid aggregates composed of minerals and other materials (volcanic glass, biological bones, rock debris, etc.) [1]. From the perspective of scientific research, the study of rocks helps to understand the geological evolution history, rock chemical composition, and petrological characteristics of a certain region. In a practical sense, the research of rocks helps to find mineral resources and water resources. Professionals can directly classify and identify rock samples. This method is mainly based on human observation and empirical classification. This method has problems such as inability to quantitatively analyze, low efficiency, greater influence by human subjective factors, high professional degree, and inability to popularize [2]. With the rise of deep learning, the possibility of automatic identification and classification of rocks has been provided [3, 4]. The application of deep learning to rock image recognition and classification has significant practical significance [5].

The identification and classification of rocks is a complex process [6]. In recent years, a large number of scholars have conducted in-depth research on the classification of rocks and achieved certain results. Lin et al. [7] used a deep learning method based on a convolutional neural network for rock recognition, and the classification accuracy rate of 15 kinds of common rock image data could reach 63%. Guojian and Peisong [8] proposed a method of rock slice image classification based on residual network, which used residual network model to automatically extract and classify the features of rock slice images. Pascual et al. [9] used a 3-layer convolutional neural network (CNN) network method to classify rock images to improve the recognition accuracy. Tian et al. [10] proposed an automatic classification method of sandstone based on SVM [11], using PCA [12] to reduce the dimension of the feature space and using SVM to obtain the relationship between the feature space and sandstone types. The test set results showed that the classification accuracy of the support vector machine classifier could reach 97.0%. Gao et al. [13] proposed a novel feature selection method-feature correlation combination (CFR). Experiments showed that feature correlation combination could effectively retain the relevant features of the data and eliminate redundant features as much as possible to improve the classification accuracy. The above studies have achieved certain results for rock image classification; however, these studies have many shortcomings and deficiencies: (1) The dataset required for training the model is large, the preparatory work is large, and too much manpower and financial resources are consumed. (2) For superdivision images, it is difficult to extract finer image information, and the quality of feature extraction is low. (3) For the problem of fine-grained image classification, it is difficult to obtain more accurate classification results.

In order to solve the problems in the above research, this paper proposes an algorithm that combines image cutting and SBV algorithm. The specific work is as follows: (1) Without adding the interference information, the data set is expanded by cutting the image to extract the more comprehensive feature information in the image as far as possible. (2) SBV classification algorithm is proposed to obtain richer complementary information to improve the classification accuracy of the deep learning network model.

## 2. Data Augmentation Technology

The deep learning algorithm model is obtained by training the neural network model, which is essentially a process of constantly adjusting parameters so that the model can map the image to the label [14]. What we need is to make the loss function of the model as low as possible. The deep learning algorithm requires a large number of datasets as input, which can make the parameters of the model fully trained and can also improve the generalization ability of the model. Therefore, it is necessary to improve the dataset of the sample [15].

### 2.1. CutMix

CutMix [16] is an image data enhancement method. First of all, it clears the pixel values of some areas of the image. Secondly, it randomly fills the pixel values of other data in the training set. Finally, we distribute the classification results according to the proportion of the filling area, thus completing the augmentation of the training set data. It has some advantages: noninformation pixels will not appear in the training process, which can improve training efficiency; it retains the advantages of data regional dropout; by requiring the model to recognize the object from the partial view and adding other pieces of sample information to the cleared area, the positioning ability of the model can be further enhanced, and so on. CutMix data augmentation map and original image and their labels are shown in Table 1.

### 2.2. Image Cutting

**Figure 1:**
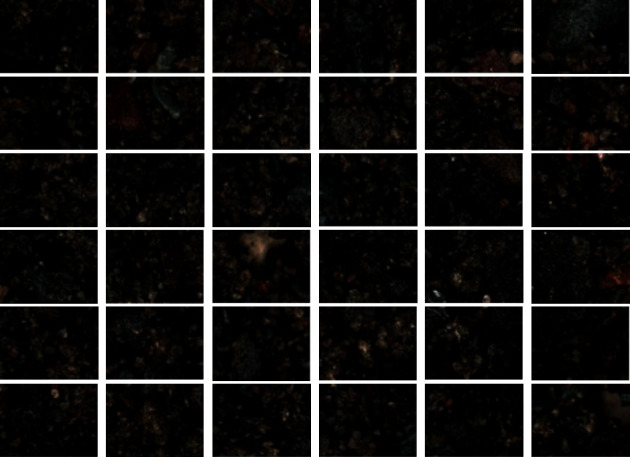
Image 6 × 6 segmentation.

The characteristic of rock image is that the local features are very similar to the global features, which is easy to think of the use of image cutting to expand the dataset to achieve the function of data enhancement. The original image pixel array is uniformly cut into *N* × *N* = *M* pictures with a smaller resolution, and the *M* pictures are put together to form a complete image. As shown in Figure 1, the image cutting when *N* is 6 is selected.

## 3. The Network Structure Design of This Paper

### 3.1. Conventional Classification Training Network Structure

**Figure 2:**
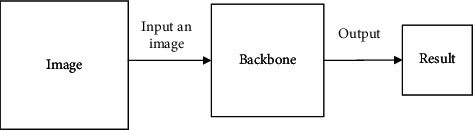
The structure of the conventional classification network.

The conventional network structure used for training is shown in Figure 2. It is generally composed of only one backbone network module. First, input the image training data into the backbone network. Next, the network output will be obtained through the calculation of the multilayer neural network. And then the difference between it and the expected output will be used to construct the loss function. After that, a suitable optimization algorithm is selected to perform gradient descent on the loss function to update the network parameters. Finally, a better network model is obtained after a certain round of iterative training.

In recent years, with the vigorous development of the big data industry, deep learning has also been advancing by leaps and bounds. Researchers have proposed a large number of classic neural network models. Among them, there are three kinds of representative models: well-designed convolutional neural networks: LeNet, GoogLeNet, SE-ResNet, and so on; networks obtained by compound model expansion method combined with neural structure search technology: EfficientNet, EfficientDet, and so on; the natural language processing (NLP) which has become a standard transformer architecture network: Vision Transformer (ViT), DeiT, and so on. To test the robustness of the new framework proposed in this paper on the rock classification problem, we chose to test it under three different types of backbone. Among them, SE-ResNet-50 [17] achieved more useful features and worked better than other same-type networks, ViT [18] transformed the image classification problem into NLP problem and achieved the state-of-the-art levels on multiple datasets, EfficientNet-B0 [19] is a model with leading speed and higher accuracy, and they are all the mainstream backbone network modules. Therefore, SE-ResNet-50 and EfficientNet-B0 were selected, respectively, as backbone network modules to perform rock image classification experiments on the new framework to validate the performance of the new model.

**Figure 3:**
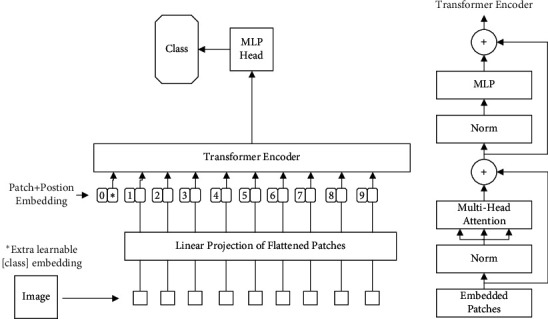
Vision Transformer network architecture.

**Figure 4:**
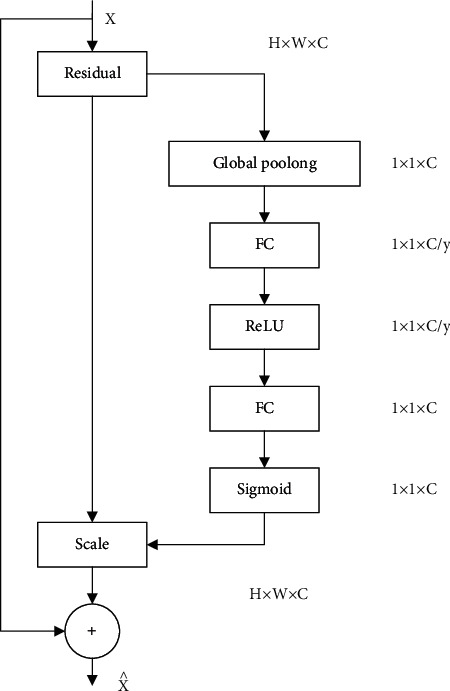
SE-ResNet network architecture.

ViT network architecture is shown in Figure 3; SE Block module is introduced based on the ResNet model to construct SE-ResNet network architecture as shown in Figure 4 and the network structure of EfficientNet-B0 is shown in Table 2.

### 3.2. Conventional Classification Prediction Network Structure

The conventional network structure used for prediction is shown in Figure 2. It is the same as the traditional network structure used for training. It is consistent with the regular classification training network structure. Input the image data to be predicted into the network, obtain the feature map of the image through the calculation of the network, and complete the classification.

The network structure in Figure 2 can get the classification result, as in formula (1), where *x* is the input image data, *y* is the classification result, *f*(*·*) is represented as the feature extractors in the backbone network, and softmax(*·*) is a normalized exponential function that acts as a classifier.

(1) $$\begin{array}{c} y=\text{softmax}\left(f\left(x\right)\right). \end{array}$$

### 3.3. Improved Classification Training Network Structure

**Figure 5:**
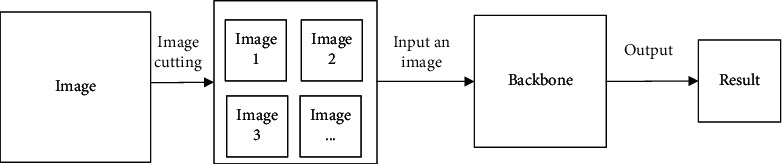
Improved classification training network structure.

The network model structure designed for training in this paper is shown in Figure 5, including two parts: image widening and trunk network. Compared with the conventional classification training network structure, the difference in this paper is to cut the image before entering the backbone network and then to input the cut image data in random order into the network training.

Among them, the image augmentation module is the image cutting method in Section 2.2, and the cutting rule is as shown in formula (2). Among them, (*A*, *B*) represents the resolution of the original image, (*A*1, *B*1) represents the resolution after cutting, “//”represents the rounding symbol, and *N* represents the number of image cuts.

(2) $$\begin{array}{c} \left(A1,B1\right)=\left(A//N,B//N\right). \end{array}$$

### 3.4. Improved Classification Prediction Network Structure

**Figure 6:**
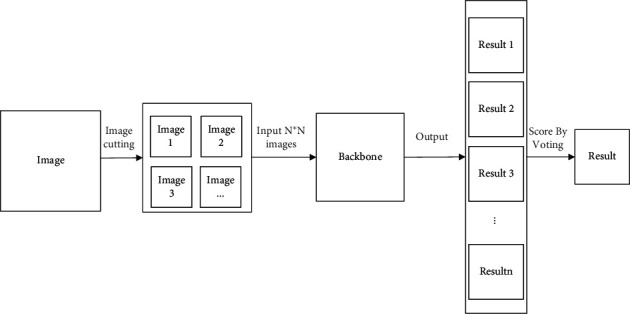
Improved classification prediction network structure.

Different from the traditional classification network, the structure of the classification prediction network model in this paper is slightly different from the structure of the classification training network model. The structure of the network model designed in this paper for prediction is shown in Figure 6. It contains three parts, namely, image augmentation, backbone network, and voting scoring. Among them, the image augmentation module, the backbone network module, and the two modules in the network model for classification training are the same. Compared with the network model of classification training, the network model structure of the classification test adds a part: score by voting module.

The K-nearest neighbor (KNN) classification algorithm [20] is very popular with clustering algorithms, whose core idea is that if most of the *K* most adjacent samples in the feature space of a sample belong to a certain category, the sample also belongs to this category and has the characteristics of the sample on this category. The method determines the category of the test sample based on the category of one or more of the nearest neighbors. The KNN method is only related to a very small number of adjacent samples in category decisions.

According to the KNN algorithm, this paper proposes scoring by voting (SBV) algorithm, whose core idea is that a category has the largest share of the classification space of the cutting image. Its mathematical expression is shown in formula (3), where max_*n*(·)_ represents the *n* value with the largest proportion, *n* represents the type coding, *i* represents the number of cuts of the image, *N* represents the total number of cuts of the image, *y*_*i*_ represents the predicted type of the *i*-th cutting graph, and *F*_*i*_^*n*^ represents that the predicted type of the *i*-th graph is *n*.

(3) $$\begin{array}{c} O=\underset{n}{\max}\left(\sum_{i=N\times N}\left(F_{i}^{n}\right)\right), \end{array}$$

(4) $$\begin{array}{c} F_{i}^{n}=\left\{\begin{array}{cc} 1, & \text{if }{\text{y}}_{i}=n,\\ 0, & \text{else.} \end{array}\right. \end{array}$$

## 4. Experimental Comparison and Analysis

### 4.1. Data Preparation

The experimental data in this paper are to use industrial cameras to take pictures of cuttings and core samples at the logging site and to take white light pictures in a dark box. The experimental categories cover four categories of mudstone, coal, fine sandstone, and siltstone, including dark gray mudstone, black coal, gray fine sandstone, light gray fine sandstone, dark gray silty mudstone, gray-black mudstone, and gray argillaceous siltstone, with a total of 315 rock samples. The resolution of the picture is divided into two categories (4096, 3000) and (2448*∗*2048). The superresolution image paves the way for the cutting method of this paper. The number of various rock samples is shown in Table 3.

### 4.2. Parameter Setting and Experimental Evaluation Index

The algorithm in this paper runs on a computer with Intel (R) Core i5-6500 CPU and GPU RXT 1660, the operating system is Windows 10, the programming language is Python, the open-source deep learning framework used is PyTorch, and some parameter settings are shown in Table 4.

For the rock image classification problem, the label smoothing [21] cross-entropy loss function is introduced to suppress overfitting, and the momentum stochastic gradient descent (SGD) optimizer is selected to update the weight parameters of the network. The initial value of the learning rate is set to 0.01. At the same time, the exponential decay method is used to adjust the learning rate. The minimum learning rate is limited to 1*e* − 5. The training rounds of each experiment are 500, the CutMix data augmentation method are used in the first 300 rounds, and the CutMix data augmentation method are removed in the next 200 rounds. After reading the picture, the data feature sizes of the input nodes are all 224, 224, and 3. The value of *α* in the CutMix data augmentation method is set to 1 so that the value of *λ* is a random value between (0, 1) numbers. The classifier conceived is certainly valid for images that are not affected by uncertainties and inaccuracies. Then, in these cases, it would be necessary to carry out a fuzzy preprocessing of the images [22–24]. Therefore, we employed Gaussian filtering in the image fuzzy preprocessing phase.

The experiment used classification accuracy as an indicator of the evaluation results to analyze the performance of the algorithm. Classification accuracy [25] is one of the most commonly used image classification evaluation indicators, which is defined as the proportion of the correct number of images predicted by the model to the total number of images predicted by the model, as shown in formula (5), where num_*R*_ represents the number of images correctly predicted by the model and *num*_*A*_ represents the total number of images predicted by the model.

(5) $$\begin{array}{c} \text{Acc}=\frac{{\text{num}}_{R}}{{\text{num}}_{A}}. \end{array}$$

### 4.3. Experimental Results

**Figure 7:**
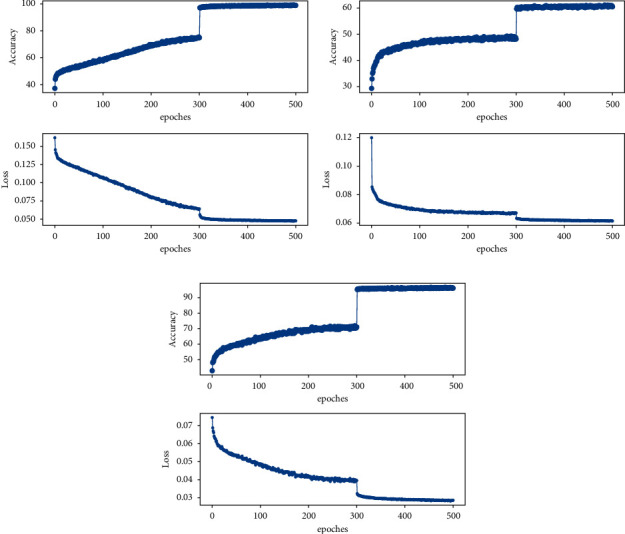
The training process using image cutting. (a) SE-ResNet-50-image cutting; (b) ViT-image cutting; (c) EfficientNet-b0-image cutting.

The improved classification training network was used in this paper to experiment on the rock image dataset, as shown in Figure 7, which are the accuracy and loss function change graphs of the SE-ResNet-50, ViT, and EfficientNet-B0 models using the image cutting method. It can be seen from Figure 7 that as the iteration progresses, the loss functions of the SE-ResNet-50, EfficientNet-B0, and ViT networks steadily decrease, and the classification accuracy of the training set steadily increases. After 300 iterations, the loss function of each network is still declining. The accuracy of the training set of SE-ResNet-50 and EfficientNet-B0 is close to 100%, and the accuracy of the training set of the ViT network has reached 60%. After 300 iterations, the loss function of each network is still declining. The accuracy of the training set of SE-ResNet-50 and EfficientNet-B0 is close to 100%, and the accuracy of the training set of the ViT network has reached 60%.

Table 5 shows the comparison of ablation experiment results of different algorithms. Among them, the classification accuracy rates of the SE-ResNet-50, ViT, and EfficientNet-B0 models that do not incorporate additional methods under the test set are 40.625%, 50.0%, and 31.25%, respectively. The classification accuracy of the SE-ResNet-50, ViT, and EfficientNet-B0 models of the fusion image cutting algorithm under the test set is 28.125%, 43.75%, and 34.375%, respectively. The classification accuracy rates of the SE-ResNet-50, ViT, and EfficientNet-B0 models that incorporate SBV algorithm under the test set are 71.875%, 56.25%, and 75.0%, respectively. The classification accuracy rates of SE-ResNet-50, ViT, and EfficientNet-B0 models that simultaneously integrate image cutting and SBV algorithms under the test set are 75.0%, 68.75%, and 75.0%, respectively.

## 5. Discussions

**Figure 8:**
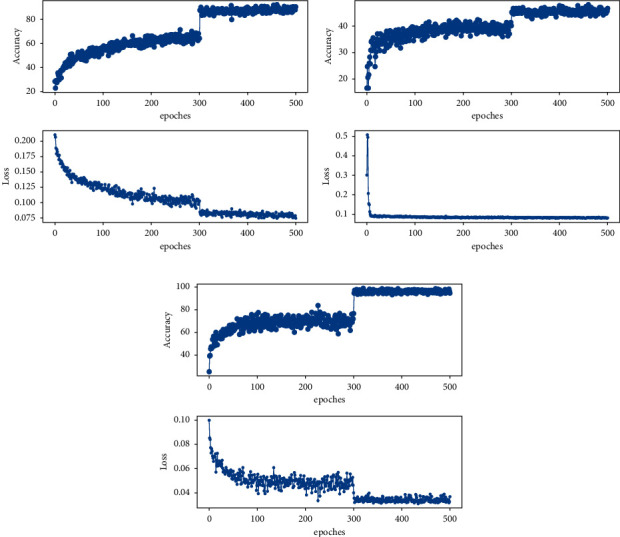
The training process of image cutting. (a) SE-ResNet-50; (b) ViT; (c) EfficientNet-B0.

Experiments were performed on the rock image dataset using a conventional classification training network, as shown in Figure 8, respectively, for the accuracy and loss function plots of the SE-ResNet-50, ViT, and EfficientNet-B0 deep learning model in the training set without image cutting. As can be seen from Figure 8, with the increasing number of iterations, the loss function shock of SE-ResNet-50 and EfficientNet-B0 network decreases, the classification accuracy shock of the training set increases, and the difference is that the loss function of ViT network decreases rapidly and fluctuates violently after rapid improvement. After the images of the three networks were iterated up to 300 times, the loss function and accuracy of the training set changed significantly. In 300–500 iteration rounds, the loss function and accuracy of the SE-ResNet-50 and EfficientNet-B0 networks tend to be stable and the accuracy of the training set of the two has reached about 90%, but the accuracy of the training set of the ViT network only has reached about 45%. The accuracy and loss function graphs of the training set of each model in Figures 7 and 8 have a large change after 300 times. This is because of the impact of CutMix data enhancement closure [26] and the high accuracy of the training set indicates that the deep learning network can fit the rock images of the training set. Compared with Figure 8, the curve in Figure 7 is smoother. After 300 iterations, the loss function of the training set is still declining, the decline is lower, and the classification accuracy of the training set is higher. The accuracy of SE-ResNet-50 and EfficientNet-B0 networks has reached almost 100%, and the accuracy of ViT has reached 60%. It can be seen that the image cutting method proposed in this paper can improve the accuracy of the network model to a certain extent.

The experimental results in Table 5 show that the accuracy of the SE-ResNet-50 and ViT model using the image cutting method decreased by 12.5% and 6.25%, respectively, and the EfficientNet-B0 model decreased by 3.125%, indicating that the use of image cutting to increase the dataset alone does not improve the classification accuracy of small-sample fine-grained images. The accuracy of the SE-ResNet-50, ViT, and EfficientNet-B0 models using SBV algorithm alone improves by 31.25%, 6.25%, and 43.75%, respectively, indicating that the SBV algorithm can obtain more messages to help classification and effectively improve the classification accuracy of fine-grained images with fewer samples. SE-ResNet-50, ViT, and EfficientNet-B0 models using both the image cutting method and the SBV algorithm were 75%, 68.75%, and 75%, respectively, which improved 34.375%, 18.75%, and 43.75%, respectively, over the models without any method, demonstrating the effectiveness and robustness of the algorithm.

To further analyze the rock image classification performance of the algorithm in this paper, the algorithm in this paper is compared with the commonly used VGG (Bai, 2019) [27], AlexNet (Yang, 2021) [28], PCA-SVM (Tian, 2019) [10], and Bilinear CNN (Lin *T*, 2015) [29] and other algorithms are compared on the rock image dataset of this paper. The classification results are shown in Table 6. It can be seen from Table 6 that the algorithm in this paper has higher accuracy on the rock image dataset. The image classification accuracy of the algorithm in this paper is 75%, which is 28.1255%, 53.125%, 34.375%, and 18.75% higher than that of the VGG, AlexNet, PCA-SVM, and Bilinear CNN algorithms, respectively. It also shows that the algorithm proposed in this paper has excellent performance.

When applying deep learning to the classification of a small number of fine-grained rock images, through the test, there are some findings:

1. The experimental results of the test set classification show that our method can effectively alleviate the lack of data, which makes it difficult for the model to extract sufficient feature information. This method avoids the shortcomings of traditional methods, such as low efficiency and susceptibility to subjective factors, which rely on professional experience classification.
2. The performance comparison of ResNet-50, ViT, and EfficientNet-B0 models before and after modification shows that when the image cutting method is used alone, the performance of the model sometimes decreases, but when the SBV algorithm is used at the same time, the performance of the model is greatly improved.
3. The method proposed in this paper can be used for rock classification in interstellar exploration. The steps are as follows. First, the camera collects the rock image in real time to obtain the rock image to be measured and then uses the microprocessor to preprocess the rock image to be measured and input it into the trained model for prediction. Finally, the classification result is directly transmitted back through the communication system. There is no need to go through sample collection. This program can realize artificial intelligence-based interstellar exploration.

## 6. Conclusions and Future Work

Aiming at the problem of fine-grained rock classification with few samples, this paper proposes a data augmentation method to alleviate the problem of lack of sample data by cutting the image without adding additional data samples and SBV algorithm based on deep learning network is proposed to construct a new classification prediction network structure. The experimental results show that, on the rock image dataset in this paper, the classification accuracy of the three networks of SE-ResNet-50, ViT, and EfficentNet-B0 is 40.625%, 50.0%, and 31.25%, respectively. Using the image cutting method proposed in this paper and the SBV algorithm network, the classification accuracy of 32 images obtained by the test is 75%, 68.75%, and 75%. The results show that the method proposed in this paper has a significant improvement in the accuracy of image classification. The original algorithm has increased by 34.375%, 18.75%, and 43.75% respectively. It also proves that the neural network replaces the traditional rock sample identification method with good application value and has certain research value for the identification and classification of rocks. Deep learning technology requires a large number of datasets and clear images. The method proposed in this paper can greatly improve the accuracy of the algorithm in the case of a small number of datasets. However, there are still two research directions that can be further studied in the future: (1) Deep learning is a black-box model, and its interpretability needs further research. (2) Open-source rock datasets are lacking. In other cases, the robustness of the method in this paper is unknown. The establishment of open-source fine-grained rock datasets is a significant study.

## Figures and Tables

**Table 1:** CutMix data augmentation map and original image display.

	Original image	CutMix
Image	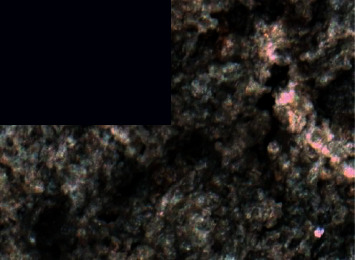	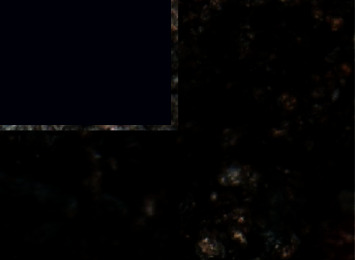

Label	Gray fine sandstone 1.0	Gray fine sandstone 0.25 Dark gray mudstone 0.75

**Table 2:** Network structure of EfficientNet-B0.

Stage	Operator	Resolution	Number of channels	Number of layers
*i*	*F*_*i*_	*H*_*i*_ × *W*_*i*_	*C*_*i*_	*L*_*i*_
1	Conv3 x 3	224 × 224	32	1
2	MBConv1, k3 x k3	112 × 112	16	1
3	MBConv6, k3 x k3	112 × 112	24	2
4	MBConv6, k5 x k5	56 × 56	40	2
5	MBConv6, k3 x k3	28 × 28	80	3
6	MBConv6, k5 x k5	14 × 14	112	3
7	MBConv6, k5 x k5	14 × 14	192	4
8	MBConv6, k3 x k3	7 × 7	320	1
9	Conv1 x 1&Pooling&FC	7 × 7	1280	1

**Table 3:** Number of seven types of rocks.

Type of rock	Number of rocks
Dark gray mudstone	75
Black coal	21
Light gray fine sandstone	85
Dark gray silty mudstone	40
Gray-black mudstone	30
Gray argillaceous siltstone	46
Gray fine sandstone	18

**Table 4:** Training parameter table.

Parameter name	Numerical value
Batch-size	10
Momentum	0.9
Seed	1
Decay_rate	0.98
Dropout	0.1
Epoch	500
*N*	6

**Table 5:** Comparison of classification accuracy of different algorithms under the test set.

Method	Model	Accuracy (%)
Original	SE-ResNet-50	40.625
	ViT	50.0
	EfficentNet-B0	31.25

Image cutting	SE-ResNet-50	28.125
	ViT	43.75
	EfficentNet-B0	34.375

SBV	SE-ResNet-50	71.875
	ViT	56.25
	EfficentNet-B0	75.0

Image cutting + SBV	SE-ResNet-50	75.0
	ViT	68.75
	EfficentNet-B0	75.0

**Table 6:** Comparison of the classification accuracy of the algorithm in this paper and other algorithms on the test set.

Method	Accuracy (%)
VGG (Bai, 2019)	46.875
AlexNet (Yang, 2021)	21.875
PCA-SVM (Tian, 2019)	40.625
Bilinear CNN (Lin T, 2015)	56.25
Ours	75

## Data Availability

The data used to support the findings of this study are available from the corresponding author upon request.
